# Surgical‐ and implant‐related factors and onset/progression of peri‐implant diseases: An AO/AAP systematic review

**DOI:** 10.1002/JPER.24-0083

**Published:** 2025-06-09

**Authors:** Alberto Monje, Shayan Barootchi, Paul S. Rosen, Hom‐Lay Wang

**Affiliations:** ^1^ Department of Periodontology and Oral Medicine University of Michigan Ann Arbor Michigan USA; ^2^ Department of Periodontology Universitat Internacional de Catalunya Barcelona Spain; ^3^ Department of Periodontology University of Bern Bern Switzerland; ^4^ Department of Periodontology Harvard School of Dental Medicine Boston USA; ^5^ Department of Periodontology Rutgers University School of Dental Medicine Piscataway USA

**Keywords:** complication endosseous implant, dental implant, mucositis, peri‐implant disease, Peri‐implantitis

## Abstract

**Background:**

The objective of this systematic review was to shed light on the importance of surgical‐ and implant‐related factors on the onset/progression of peri‐implant diseases. This systematic review provides an evidence‐based overview on the prevention of peri‐implant diseases for the Academy of Osseointegration (AO)/American Academy of Periodontology (AAP) Consensus Conference held in August 14–16, 2024 in Oakbrook, IL, USA.

**Methods:**

Systematic screening of electronic sources was performed to identify clinical studies reporting on the impact of surgical‐ and implant‐related factors on peri‐implant diseases. To fulfil the inclusion criteria, a composite clinical and radiographic case definition for peri‐implant mucositis and/or peri‐implantitis had to be reported. Prevalence or incidence (%) of peri‐implant diseases was extracted at patient‐ and implant‐level. Moreover, odds/hazard ratios were collected to explore potential associations.

**Results:**

Overall, 34 articles were included in the qualitative synthesis. Of these, 21 explored surgical‐related factors (*N*_patients _= 2752, *N*_implants _= 7591), while 13 reported implant‐related factors (*N*_patients _= 1192, *N*_implants _= 4072). Given the high heterogeneity across the studies, only qualitative assessment was performed. Clinical evidence proved that surgical‐related factors, in particular inadequate implant position and, to a lower extent, implants placed in regenerated bone, are more prone to exhibit peri‐implantitis, but not peri‐implant mucositis. With regards to specific implant‐related factors, insufficient evidence, did not allow for exploring specific associations with the onset/progression of peri‐implant diseases. However, it was found that a short distance from the prosthetic margin to crestal bone during implant placement may be a predisposing factor for peri‐implantitis.

**Conclusion:**

Clinical evidence linking surgical‐ and implant‐related factors and peri‐implant diseases is sparsely reported in the literature. Nevertheless, it appears that implant malposition plays a crucial role on the onset/progression of peri‐implantitis.

**Plain Language Summary:**

The objective of this systematic review was to shed light on the importance of surgical‐and implant‐related factors on the onset/progression of peri‐implant diseases. Overall, 33 articles were included in the qualitative synthesis. Of these, 21 explored surgical‐related factors. Given the high heterogeneity across the studies, only qualitative assessment was performed. Clinical evidence proved that surgical‐related factors, in particular inadequate implant position and, to a lower extent, implants placed in regenerated bone, are more prone to exhibit peri‐implantitis, but not peri‐implant mucositis. With regards to specific implant‐related factors, insufficient evidence, did not allow for exploring specific associations with the onset/progression of peri‐implant diseases. However, it was found that a short distance from the prosthetic margin to crestal bone during implant placement may be a predisposing factor for peri‐implantitis. All in all, implant malposition plays a crucial role on the onset/progression of peri‐implantitis.

## 1 INTRODUCTION

Peri‐implant diseases are regarded as being biofilm‐mediated inflammatory entities.^1^ Despite shared features with periodontal diseases in both their etiology and risk factors, there are notable differences concerning the pathogenesis and pattern of progression. Peri‐implant mucositis is diagnosed when the inflammation is confined within the soft tissues surrounding the dental implant. In other words, no progressive bone loss beyond the physiological bone modeling/remodeling has occurred as response to the bacterial insult.^1^ Prevention measures of peri‐implantitis can be implemented with high success.^2^, ^3^ Although experimental peri‐implant mucositis has been shown to be partially reversible, its resolution may take 2× longer when compared with that of experimental gingivitis.^4^ If mucositis is not arrested, peri‐implantitis may then be the consequence.^1^ Peri‐implantitis, in contrast, displays progressive marginal bone loss and may compromise the longevity of dental implants, while increasing the susceptibility to experience life‐threatening conditions.^5^ Peri‐implantitis lesions are more than twice as large as those seen with periodontitis.^6^ In addition, peri‐implantitis lesions contain a higher percentage of plasma cells, macrophages, and neutrophils, and are more highly vascularized lateral to the cellular infiltrate compared with periodontitis sites.^6^ This would suggest that peri‐implantitis might be more destructive and at a faster rate than periodontitis.

The evidence is strong for bacteria being the etiology for peri‐implant diseases.^7^ In fact, disease onset and progression are associated with qualitative and quantitative differences in the microbiome when compared with peri‐implant health.^8^ Data from reports indicate that peri‐implantitis is a heterogeneous mixed infection including periodontopathic organisms such as *Tannerella forsythia*, *Prevotella intermedia*, *Fusobacterium nucleatum*, *Porphyromonas gingivalis*, uncultivable anaerobic gram+ and gram−rods, and enteric rods and *Staphylococcus aureus*.^8^, ^9^, ^10^, ^11^ It is important to note that the peri‐implant microbiome is richer and more heterogeneous than that of its periodontal counterpart.  In fact, it was demonstrated that <50% of all species between their biofilms, and 85% shared <8% of abundant species between a tooth and an implant.^10^ Therefore, even though the infectious nature of peri‐implant diseases is noticeable, the periodontal and peri‐implant microbiomes represent different ecosystems.

A feature that must be highlighted regarding peri‐implantitis, is its site‐specificity when the prevalence is assessed.^12^ This means that, in susceptible individuals, there might be sites more prone to exhibit this entity when compared with others in patients restored with multiple dental implants. This points towards the significance of local factors that may predispose/precipitate plaque accumulation and that may impact the immune response in the prevention and management of peri‐implant disorders.^13^, ^14^ For instance, data derived from a survey suggested that implant position (too buccal), soft tissue thickness (thin) and buccal band of keratinized mucosa (deficient) were contributors to peri‐implantitis.^15^ Others have even speculated that peri‐implantitis may represent an inflammatory disease led by bacterial challenge, that is, a “man‐made” disease triggered by local factors that drive towards a foreign body reaction.^16^ Thereupon, it seems that site‐specific conditions favor the development of peri‐implant diseases. Accordingly, the objective of this systematic review is to shed light on the importance of surgical‐ and implant‐related factors on the onset/progression of peri‐implant diseases.

Therefore, the objectives of the present systematic review were:

o What is the impact of surgical‐related factors (i.e., any possible relevant component, for example, implant position—bucco‐lingual, apico‐coronal‐, surgical technique—flapped, flapless, guided, free‐handed, surgical stage—immediate, early, late‐, simultaneously/staged grafting procedures, implant insertion torque) of dental implants on the onset/progression of peri‐implant disease in human clinical trials with ≥12‐month follow‐up?
o What is the effect of implant‐related factors (implant macro‐design—threaded, parallel‐wall, tapered, tissue‐ or bone‐level, implant micro‐design—implant roughness characteristics, hybrid design, or implant system of implants on the onset/progression of peri‐implant diseases in human clinical trials with ≥12‐month follow‐up?

## 2 MATERIALS AND METHODS

### 2.1 Protocol development

The protocol of this review was previously registered in the International Prospective Register of Systematic Reviews (PROSPERO) with the identification code CRD501977. This review fully adhered to the guidelines of the Preferred Reporting Items of Systematic Reviews and Meta‐Analyses (PRISMA) statement.^17^

The following PECOS framework is used to guide the inclusion and exclusion of studies for the aforementioned focused questions:

- Population (P): Patients (human adults) presenting with at least one dental implant previously in function, reported with the condition mucositis or peri‐implantitis, as diagnosed, and specified according to the case definition by the American Academy of Periodontology/European Federation of Periodontology 2017 World Workshop^1^ or a clear composite criterial based on clinical and radiographic data.
- Exposure (E): Any pristine or modified feature that relates to the above‐mentioned local confounders (surgical‐ and/or implant‐related factors)
- Comparison (C): Any pristine feature that related to the above‐mentioned local confounders (surgical‐ and/or implant‐related factors)
- Outcomes (O): For inclusion, studies must provide the rate (incidence/prevalence/progression) of peri‐implant diseases compared or not with peri‐implant health. Other secondary outcomes that will be extracted from included studies are as follows:
  - Odds (OR) or hazard ratio (HR) for mucositis
  - Odds (OR) or hazard ratio (HR) for peri‐implantitis
  - Concluding remarks
- Study Design (S):
  - Human clinical trials including randomized clinical trials, prospective or retrospective cohort and case control studies with implants that have been restored at least ≥12 months after the event of peri‐implantitis and ≥10 patients will be included. No language restriction is aimed at being applied if the study is published in a peer‐reviewed journal.

### 2.2 Eligibility criteria

Studies (randomized clinical trials [RCTs], comparative prospective and retrospective cohorts, cross‐sectional or case series) that recruited subjects where dental implants were opted to be placed aimed at restoring function and/or esthetics and report on surgical‐ factors as confounders of peri‐implant health/disease. To assess the effect of implant‐related factors on peri‐implant diseases only comparable studies were included. A minimum number of 10 patients (overall) and a postoperative follow‐up period of at least 12 months after final prosthesis delivery were required for eligibility. Included studies must be adhered to a definition of peri‐implant disease.

### 2.3 Search strategy

One independent reviewer (AM) performed the screening and read the title and abstract of the entries obtained from the literature search. After completing the screening process, another reviewer was consulted (SB) to assess the full‐text version of potentially eligible studies and established a final article selection. Disagreements between the reviewers were resolved by open discussion. If no consensus could be reached, a third author (HLW or PSR) was consulted. Any missing information that could contribute to the systematic review was requested from the corresponding author(s) via e‐mail.

### 2.4 Information sources

An electronic search of two databases (MEDLINE via PubMed and the Cochrane Library of the Cochrane Collaboration) was conducted for studies published up to October 2023 (included), without language or year restrictions. The search strategy combined MeSH terms and text words with Boolean operators (OR, AND) filtered by “humans” and “animals” and sorted according to the most recent publications. Table S1 presents the screening strategies and the results for each of the factors explored (see Table S1 in online *Journal of Periodontology*). Moreover, the Cochrane database and the Grey Literature Database were screened for unpublished papers in the New York Academy of Medicine Grey Literature in accordance with the AMSTAR checklist. The list of references of the included studies and related review articles were further screened to check for additional relevant studies.

### 2.5 Data extraction

The following data were extracted and recorded in duplicate by two independent reviewers: (1) citation and year of publication; (2) experimental group; (3) sample size; (4) factor related to peri‐implant diseases; (5) case definition of peri‐implant diseases; (6) rate of mucositis at patient‐ and implant level; (7) rate of peri‐implantitis at patient‐ and implant‐level; (8) odds/hazard ratio and; (9) concluding remarks

### 2.6 Data synthesis

Qualitative analysis was aimed at being performed given the high heterogeneity across the studies to perform meta‐analysis. Sample characteristics and the impact of surgical‐ and implant‐ related factors on peri‐implant diseases were comprehensively retrieved from the included publications and presented in tables. The data are being synthetized applying a narrative approach.

### 2.7 Risk of bias assessment

The risk of bias analyses of the included investigations was independently performed by two examiners using the Cochrane Collaboration's tool for assessing the risk of bias in RCTs.^18^ RCTs were categorized as low, with some concerns or high risk of bias. The National Institute of Health (NIH) quality assessment was used for case series, for cohort and for cross‐sectional studies.^19^ Disagreement between reviewers was resolved by open discussion. In case no agreement could be achieved, the final decision was made by a third reviewer.

## 3 RESULTS

### 3.1 Screening

**FIGURE 1:**
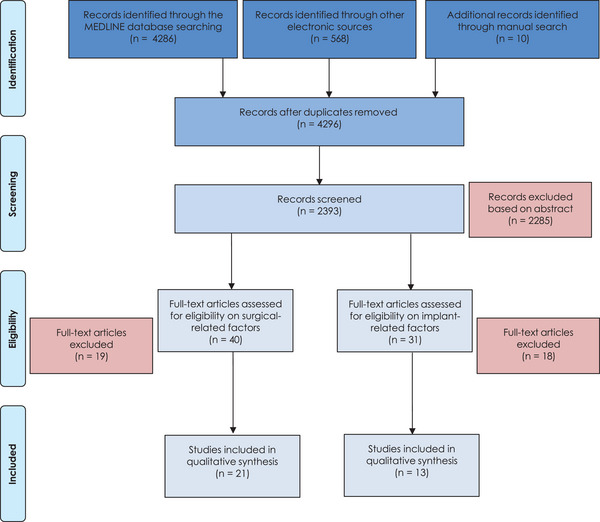
Flowchart of the screening process.

The PRISMA flowchart for literature selection is depicted in Figure 1. In summary, 4296 records were identified after duplicates were removed. Seventy‐one of these records were assessed for full‐text. Overall, 34 were included in the qualitative synthesis. Of these, 21 explored surgical‐related factors (Table 1), while 13 implant‐related factors (Table 2). The most frequent reason for exclusion, based on the full‐text evaluation, was no data concerning the prevalence or case definition of peri‐implant diseases. Due to the significant heterogeneity among the available articles, as well as the inherent methodological variabilities and potential biases among the included study designs, the selected studies were analyzed descriptively.

**TABLE 1:** Surgical‐related factors on the onset/progression of peri‐implant disease.

Author (year)	Surgical‐related factor	Study design	Study period (years)	Patients (*n*)	Implants (*n*)	Adjunctive intervention	Case definition of mucositis	Case definition of peri‐implantitis	Mucositis rate (%) at patient level	Peri‐implantitis rate (%) at patient level	Mucositis rate (%) at implant level	Peri‐implantitis rate (%) at implant level	OR/HR for mucositis	OR/HR for peri‐implantitis	Concluding remarks	Risk of bias
Bienz et al. (2023)	Soft tissue grafting	Retrospective case‐control	12	8	8	Connective tissue graft	World Workshop AAP/EFP (2017)	World Workshop AAP/EFP (2017)	33.3	0	33.3	0	NR	NR	The use of connective tissue graft staged to implant placement in the esthetic zone may not be relevant in the onset/progression of peri‐implant diseases	10/14
				10	10	Control			85.7	0	85.7	0	NR	NR		
Buonocunto et al. (2023)	Alveolar ridge preservation	Multicenter cross‐sectional	2.5–15	51	61	Alveolar ridge preservation	World Workshop AAP/EFP (2017)	World Workshop AAP/EFP (2017)	45.1	5.9	37.7	8.2	NR	NR	Implants placed in sites that previously received bone grafting for ridge preservation display favorable rates of peri‐implant health in the long‐term	10/14
Canullo et al. (2016)	Implant position	Retrospective case‐control	≥2	56	332	Inadequate implant position	NR	PPD ≥ 4 mm + BOP/SUP + MBL ≥3 mm	NR	NR	NR	12	nr	48.2	The rate of implant position related peri‐implantitis was 47% and failed bone reconstruction related 58.2% from all peri‐implantitis cases. Peri‐implantitis is very often associated to surgical‐related factors	6/9
	Bone regeneration					Failed bone reconstruction			NR	NR		13.8	NR	2.3		
Canullo et al. (2017)	Implant position	Retrospective case‐control	7.4	53	231	Inadequate implant position	NR	PPD ≥ 4 mm + BOP/SUP + MBL ≥3 mm	NR	NR	NR	17.1	NR	NR	The rate of implant malposition related peri‐implantitis was 17.1% from all the peri‐implantitis cases. Prosthetic‐related factors might be more decisive than surgical‐related factors	6/9
Corbella et al. (2023)	Implant position	Retrospective cohort	6.4	90	180	Inadequate implant position	NR	MBL ≥ 3 mm	NR	NR	NR	6.1	NR	NR	The rate of peri‐implantitis was from all the implant malposition was 21.2%. Implant malposition is not associated with peri‐implantitis. Emergence profile ≥45° is correlated to an increased risk of peri‐implantitis	8/14
Jemt et al. (2017)	Surgical stages	Retrospective cohort	≥5	1017	3082	Surgical technique (immediate vs. 1 vs. 2 stage)	NR	BOP/SUP + >0.6 mm	NR	NR	NR	NR	NR	1.88	Peri‐implantitis seemed to be more frequently associated to immediate implants in the posterior mandible	8/14
	Number of implants					Number of implants				NR	NR	NR	NR	1.32		
Naeini et al. (2023)	Guided implant surgery	Prospective cohort	9.1	16	69	Guided implant surgery	NR	World Workshop AAP/EFP (2017)	NR	18.7	NR	6.3	NR	NR	Flapless guided implant surgery seems effective in the long‐term to prevent peri‐implantitis	8/14
Daubert el al. (2015)	Implant diameter	Cross‐sectional	10.9	96	225	Wide implant diameter	BOP and/or inflammation	PPD ≥ 4 mm + BOP/SUP + MBL ≥2 mm	NR	NR	NR	NR	NR	1.6	Factors such as diabetes and periodontal disease are indicators of peri‐implantitis	9/14
Kolerman et al. (2023)	Bone regeneration/ surgical stage	Retrospective cohort	8	42	75	Simultaneous bone regeneration and immediate implant placement	World Workshop AAP/EFP (2017)	AAP/EFP (2017)	45	20.7	NR	NR	NR	NR	Immediate placement and restoration of the mandibular incisors demonstrated effective	8/14
Kütan et al. (2014)	Implant position	Randomized clinical trial	3	28	28	Subcrestal implant position (1 mm)	NR	MBL ≥1 thread	0	0	0	0	NR	NR	More marginal bone resorptions occurred after the third year of loading in implants placed 1 mm below bone level. However, the reduced marginal bone level was not associated with biological complications	Low
				28	28	Crestal implant position	NR		0	0	0	0	NR	NR		
Kumar et al. (2018)	Implant position	Retrospective case control	≥5	86	222	Apico‐coronal implant position	NR	PPD ≥ 4 mm + BOP/SUP + MBL ≥ 2 mm	NR	NR	NR	NR	NR	8.5	Mean platform depth of 6 mm or more from adjacent tooth increases 8.5x the likelihood of peri‐implantitis	9/14
Mameno et al. (2018)	Bone regeneration	Retrospective cohort	≥5	477	1420	Guided bone regeneration	NR	BOP/SUP + MBL≥1 mm	NR	NR	NR	NR	NR	0.7	None of the surgical‐related factors assessed proved being associated with peri‐implantitis	9/14
	Surgical stages					Surgical technique (immediate vs. 1 vs. 2 stage)			NR	NR	NR	NR	NR	1.1	Factors such as plaque control, implants in the maxilla and less occlusal support demonstrated being indicators of peri‐implantitis while surgical‐related factors failed to yield significance	
	Implant diameter					Wide implant diameter			NR	NR	NR	NR	NR	1		
	Implant length					Long implant length			NR	NR	NR	NR	NR	0.9		
Obreja et al. (2021)	Soft tissue grafting	Cross‐sectional	6.1	19	29	Connective tissue graft	World Workshop AAP/EFP (2017)	World Workshop AAP/EFP (2017)	42.1	5.3	44.8	3.4	NR	NR	Simultaneous soft‐tissue grafting using connective tissue graft had a beneficial effect on the maintenance of peri‐implant health	8/14
				36	55	Control			52.8	13.9	52.7	9.1	NR	NR		
Obreja et al. (2021)	Implant position	Cross‐sectional	1–26	200	657	Subcrestal implant position (1‐3 mm)	World Workshop AAP/EFP (2017)	World Workshop AAP/EFP (2017)	66.5	15	62.6	7.5	NR	NR	Peri‐implant mucositis was a common finding in sub‐crestally positioned implants	9/14
Obreja et al. (2022)	Surgical stage	Cross‐sectional	3.9	44	57	Immediate implant placement and provisionalization	World Workshop AAP/EFP (2017)	World Workshop AAP/EFP (2017)	45.5	0	45.6	0	NR	NR	Immediate implant placement with an immediate provisional restoration was associated with peri‐implant tissue stability and peri‐implant health in the medium‐ and long‐term follow‐up	8/14
Ortiz‐Echeverri et al. (2023)	Surgical stages	Cross‐sectional	1–18	155	279	Immediate implant placement	World Workshop AAP/EFP (2017)	World Workshop AAP/EFP (2017)	NR	NR	13.4	17.2	NR	2.3	Immediate implant placement, implants placed in deficient sites and implants ≤11 mm showed a significantly higher rate of peri‐implantitis	8/14
	Alveolar bone dimension					Deficient alveolar width			NR	NR	61.1	68.8	NR	2.9		
	Implant length					Implant length ≤ 11 mm			NR	NR	NR	NR	NR	2.4		
Paolantoni et al. (2024)	Implant position	Retrospective cohort	5	27	63	Subcrestal implant position (1 mm)	World Workshop AAP/EFP (2017)	World Workshop AAP/EFP (2017)	19	19	19	19	NR	NR	Apico‐coronal implant position did not show to influence significantly on the rate of biological complications	9/14
				21	43	Crestal implant position			25.6	32.6	25.6	32.6	NR	NR		
Parvini et al. (2023)	Surgical stage	Cross‐sectional	2–10	47	64	Immediate implant placement and immediate loading	World Workshop AAP/EFP (2017)	World Workshop AAP/EFP (2017)	57.5	4.2	48.5	3	NR	NR	Immediate implant placement and loading demonstrated effective with low rate of peri‐implantitis	8/14
Schropp et al. (2013)	Surgical stage	Randomized clinical trial	10	16	16	Early implant placement (10 days)	BOP	BOP/SUP + MBL≥1 mm compared with baseline	64	0	64	0	NR	NR	Peri‐implant diseases were not associated to the implant placement stage in the long‐term	Low
				20	20	Delated implant placement (3 months)	BOP		94	2.1	94	2.1	NR	NR		
				17	17	Late implant placement (17 months)	BOP		53	2.1	53	2.1	NR	NR		
Schuldt‐Filho et al. (2014)	Implant position	Retrospective cohort	≥1	27	161	Distance between implants < 3 mm	NR	PPD≥4 mm + BOP/SUP + MBL ≥2 mm	NR	NR	NR	48.4	NR	8.6	Distance between implants showed being strongly associated with peri‐implantitis	8/14
Zhang et al. (2020)	Soft tissue substratum	Prospective cohort	≥2	65	159	Vertical soft tissue thickness	World Workshop AAP/EFP (2017)	AAP/EFP (2017)	NR	NR	59.9	10.8	NR	2.5	The excessive vertical soft tissue thickness around implants in patients with history of periodontitis demonstrated an adverse influence on health of the peri‐implant tissue.	10/14

Abbreviations: BOP, bleeding on probing, SUP, suppuration; MBL, marginal bone loss; PPD, pocket probing depth; NR, not reported.

The risk of bias for RCT was assessed using the Cochrane Collaboration's tool. RCTs were categorized as low, with some concerns or high risk of bias. The National Institute of Health (NIH) quality assessment was used for case series, for cohort and for cross‐sectional studies.

**TABLE 2:** Implant‐related factors on the onset/progression of peri‐implant diseases.

Author (year)	Implant‐related factor/Implant characteristics	Study design	Study period (years)	Patients (*n*)	Implants (*n*)	Specific characteristic	Case definition of mucositis	Case definition of peri‐implantitis	Mucositis rate (%) at patient level	Peri‐implantitis rate (%) at patient level	Mucositis rate (%) at implant level	Peri‐implantitis rate (%) at implant level	OR/HR for mucositis	OR/HR for peri‐implantitis	Concluding remarks	Risk of bias
Derks et al. (2016)	Implant system	Retrospective cohort	9	588	2277	AstraTech	BOP/SUP	BOP/SUP + MBL >0.5 mm	NR	NR	NR	NR	NR	3.55	Implant system, amongst other factors demonstrated being indicative of peri‐implantitis in a large cohort of patients	12/14
						Nobel Biocare			NR	NR	NR	NR	NR	3.7		
						Straumann			NR	NR	NR	NR	NR	1		
Gadzo et al. (2023)	Implant system	Randomized controlled trial	10	23	37	Astra OsseoSpeed TX (Dentsply)	World Workshop AAP/EFP (2017)	World Workshop AAP/EFP (2017)	34.8	0	29.7	0	NR	NR	Straumann Bone level implants reported higher rate of biological complications when compared to Astra OsseoSpeed implants in the long‐term assessment	Low
				20	32	Straumann Bone Level (Straumann)			52.3	4.8	50.1	6.3	NR	NR		
Gamper et al. (2017)	Implant macro‐design	Randomized controlled trial	4‐6	30	65	Tissue Level (Straumann)	PPD ≥ 5 mm + BOP >50% of sites	PPD ≥5 mm + BOP > 50% of sites + MBL ≥2 mm	7.7	10.7	5.3	7	NR	NR	Biological complications between bone ant tissue level did not reveal significant differences	Concerns
					86	Branemark Mk IIII/Mk IV (Nobel Biocare)			10	13.3	10.6	8.1	NR	NR		
Glibert et al. (2023)	Implant micro‐design	Randomized controlled trial	5	18	18	Hybrid implant surface (Southern Implants)	World Workshop AAP/EFP (2017)	World Workshop AAP/EFP (2017)	NR	0	NR	0	NR	NR	This 5‐year evaluation showed that patients that received both hybrid implants (minimally rough implant collar and moderately rough implant body) and implants with a moderately rough presented peri‐implant health with no substantial difference between them	Concerns
					18	Rough implant surface (Southern Implants)			NR	0	NR	0	NR	NR		
Guarnieri et al. (2018)	Implant micro‐design	Retrospective multi‐center study	5	74	82	Laser microtextured collar (BioHorizons)	PPD <5 mm + BOP	PPD ≥5 mm + BOP + MBL ≥2 mm	21.6	4	19.5	3.6	NR	NR	In private practice under supportive care, Implants with a laser‐microgrooved collar surface, compared to implants without a laser‐microgrooved collar surface, presented a statistically significantly lower incidence of peri‐implant diseases	12/14
					84	No laser microtextured collar (BioHorizons)			32.4	13.5	28.5	11.9	NR	NR		
Karoussis et al. (2004)	Implant macro‐design	Prospective cohort	10	89	112	ITI hollow screw (Straumann)	NR	PPD ≥ 5 mm + BOP + MBL	NR	NR	NR	10	NR	NR	ITI hollow screw implants demonstrated lower incidence of peri‐implantitis when compared to the other two designs in the long‐term	12/14
					49	ITI hollow cylinder (Straumann)			NR	NR	NR	29	NR	NR		
					18	ITI angulated hollow cylinder (Straumann)			NR	NR	NR	12	NR	NR		
Kahramanoğlu et al. (2020)	Implant macro‐design	Prospective cohort	5	30	23	Straumann Bone Level SLActive (Straumann)	Inflammation	Inflammation + MBL ≥2 mm	NR	0	NR	0	NR	NR	No differences in biological complications were noted between different implant systems	10/14
					28	Astra OsseoSpeed TX (Dentsply)			NR	0	NR	0	NR	NR		
					24	SPI (Thommen)			NR	0	NR	0	NR	NR		
Kadkhodazadeh et al. (2022)	Implant micro‐design	Retrospective cohort	2	43	60	Laser microtextured collar (BioHorizons)	World Workshop AAP/EFP (2017)	World Workshop AAP/EFP (2017)	50	17.8	40	16.7	NR	NR	Laser microtextured collar did not yield superior outcomes to laser microtextured collars in terms of peri‐implant diseases	9/14
					79	No laser microtexturing (BioHorizons)			53.6	25	40.5	12.7	NR	NR		
Marrone et al. (2013)	Implant macro‐design	Cross‐sectional	5‐18	103	83	Tissue‐level (inspecific)	PPD >5 mm + BOP	PPD >5 mm + BOP + MBL ≥2 mm	NR	NR	NR	10.8	NR	NR	Roughened implant surfaces demonstrated higher rate of peri‐implantitis when compared to moderately rough and machined implant surfaces	8/14
					181	Bone level (inspecific)			NR	NR	NR	29.3	NR	0.7		
	Implant micro‐design				94	Rough surface (inspecific)			NR	NR	NR	36.1	NR	0.2		
Parvini et al. (2022)	Implant system	Cross‐sectional	2‐6	27	27	Ankylos (Dentsply)	World Workshop AAP/EFP (2017)	World Workshop AAP/EFP (2017)	19.2	0	19.2	0	NR	NR	Both implant systems demonstrated low prevalence of biological complications in the esthetic area following immediate implant placement and restoration	9/10
				25	25	BLX (Straumann)			13.4	0	13.4	0	NR	NR		
Ravald et al (2013)	Implant system	Prospective cohort	12‐15	25	184	Astra OsseoSpeed TX (Dentsply)	Inflammation + BOP/SUP	BOP/SUP + MBL ≥2 mm	2.1	2.1	NR	NR	NR	NR	AstraTech and Branemark implants exhibited similar long‐term outcomes in terms of low incidence of biological complications	10/14
				21	187	Branemark MK III (Nobel Biocare)			2.1	0	NR	NR	NR	NR		
Renvert et al. (2012)	Implant system	Randomized controlled trial	13	19	84	AstraTech TiO surface (Dentsply)	NR	BOP/SUP + MBL ≥1 mm	NR	NR	NR	32	NR	NR	The incidence of peri‐implantitis was not affected by the different implant surfaces tested. Instead, history of periodontitis and systemic diseases exposed to higher incidence of peri‐implantitis	Concerns
				22	80	Branemark machined surface (Nobel Biocare)	NR		NR	NR	NR	39	NR	NR		
Serrano et al. (2021)	Implant micro‐design	Randomized controlled trial	1	17	17	Hybrid implant surface (TICARE)	Profuse BOP	BOP/SUP + MBL ≥2 mm	2.8	0	NR	5.6	NR	NR	The machined surface in the coronal portion of hybrid implants did not have any influence in the short‐term incidence of biological complications	Low
				18	18	Rough implant surface (TICARE)			0	0	NR	0	NR	NR		

Abbreviations: BOP, bleeding on probing; SUP, suppuration; MBL, marginal bone loss; PPD, pocket probing depth; NR, not reported, Straumann (Basel, CH), TICARE (Valladolid, Spain), Dentsply (Molndal, Sweden), Thommen (Basel, Switzerland), Southern Implants (Irene, South Africa).

The risk of bias for RCT was assessed using the Cochrane Collaboration's tool. RCTs were categorized as low, with some concerns or high risk of bias. The National Institute of Health (NIH) quality assessment was used for case series, for cohort and for cross‐sectional studies.

### 3.2 Surgical‐related factors and onset/progression of peri‐implant diseases

#### 3.2.1 Sample characteristics

The dominant study design was retrospective cohort or case series^20^, ^21^, ^22^, ^23^, ^24^, ^25^, ^26^, ^27^, ^28^, ^29^ followed by cross‐sectional studies.^30^, ^31^, ^32^, ^33^, ^34^, ^35^, ^36^ Implant position was the most frequently surgical‐related factor analyzed.^21^, ^22^, ^23^, ^26^, ^28^, ^29^, ^32^, ^37^ The follow‐up time frame reported in the included studies ranged from 1 to 26 years. Overall, 2752 patients and 7591 implants were assessed. While the most recent studies adopted the World Workshop AAP/EFP (2017) definition for peri‐implant diseases,^1^ studies prior to 2018 opted to adhere to heterogeneous case definitions that were in large part based on bleeding on probing to define mucositis and bleeding on probing and/or suppuration and marginal bone loss in a range from 0.6 to 3  mm to define peri‐implantitis (Table 1).

#### 3.2.2 Impact on peri‐implant diseases

**FIGURE 2:**
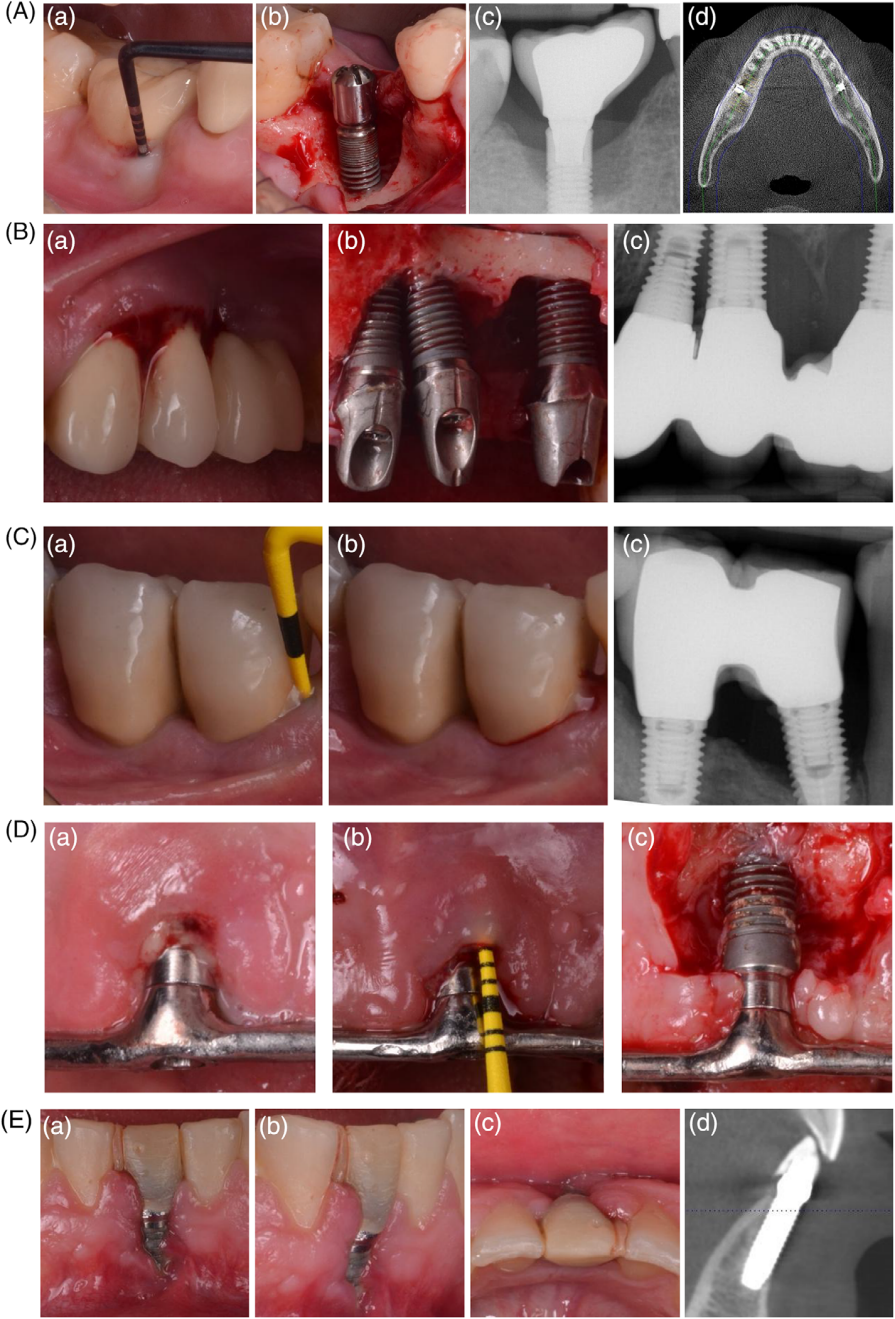
(A) Implant placed slightly towards the buccal aspect in the presence of a residual thin buccal bone. It is hypothesized that this could predispose excessive physiological bone loss that eventually leads to biofilm adhesion to the exposed implant surface. (a) Clinical image displaying peri‐implant inflammation, (b) intra‐operative image showing moderate bone loss in a crater‐like defect, (c) periapical bone loss indicating severe bone loss, (d) occlusal view in a cone‐beam computed tomography slice where it is suggested that the affected implant is slightly buccally aligned to the ideal bucco‐lingual implant position when compared with another healthy implant in the same position of the left mandibular hemiarch. (B) Malpositioned adjacent implants in a mesio‐distal direction predisposes to inadequate proximal self‐performed oral hygiene and physiologic bone loss, leading to peri‐implantitis. (a) Clinical image showing peri‐implant inflammation, (b) intra‐operative image illustrates advanced bone loss, (c) periapical x‐rays demonstrate advanced bone loss. (C) Peri‐implantitis lesions adjacent to natural teeth may have a deleterious impact upon periodontal support. (a‐b) Clinical image of clinical signs of inflammation, (c) periapical x‐ray illustrating implant proximity to the adjacent tooth where it is noted that the apical portion was injured and advanced bone loss. (D) Implants placed outside of the bony envelope often exhibit peri‐implantitis‐related dehiscence‐type bone defects (a) clinical signs of inflammation upon examination, (b) deep pockets are associated with peri‐implant pathology, (c) moderate bone loss mainly affecting the buccal aspect of the implant. (E) Implant placement in the ungrafted narrow alveolar bone may be linked with adverse dimensional changes that favor the exposure of the buccal implant surface to the oral cavity and this predisposes bacterial colonization. (a) Frontal view, (b) lateral view, (c) occlusal view, (d) cross‐sectional view exhibiting the deficient buccal bone.

In general, implant malposition in a three‐dimensional basis in relation to the alveolar bone dimension and in relation to adjacent implants/teeth demonstrated an association with peri‐implantitis (Figure 2a),^21^, ^22^, ^26^, ^29^, ^32^ but not with mucositis. Interestingly, Canullo et al. demonstrated a very strong association of implant malposition and peri‐implantitis (OR = 48.2, 95% CI = 11.4‐204.1, *p*‐value: *p* = 0.0001).^21^, ^22^ Schuldt‐Filho et al. showed that implants placed very close proximity to one another (<3  mm) were 8.6x more likely to have peri‐implantitis when compared with implants placed ≥3  mm apart (OR = 2.98, 95% CI = 1.34–7.70, *p* = 0.006) (Figure 2b).^29^ Sub‐crestal implant placement has been a subject of controversy. Obreja et al. in a cross‐sectional study demonstrated the highest rate of peri‐implant mucositis at both the patient‐ and the implant‐level were in those dental implants placed in a range from 1 to 3  mm below the osseous crest.^32^ Interestingly, this was found to be more noticeable in patients diagnosed with history of periodontitis (OR = 5.33, 95% CI = 0.22–0.94, *p* = 0.003). Moreover, Kumar et al. noticed that single‐unit implants placed ≥6  mm from the adjacent cemento‐enamel junction were 8.5× more likely to develop peri‐implantitis (OR = 8.5, 95% CI = 6.7‐32.6, *p* < 0.05).^26^ Contrary to this, two publications failed to identify associations between subcrestal implant position^28^ and implants placed in close proximity to adjacent teeth.^23^ It is noteworthy that the later study did not provide clinical evidence of disease and that implant malposition was solely analyzed using periapical x‐rays.^23^ The effect of soft tissue grafting has seemingly been controversial. Obreja et al., in a cross‐sectional study, demonstrated the significant effect of simultaneous connective tissue grafting as protective factor to prevent peri‐implant diseases.^33^ In particular, there was a statistically significant difference in the assessment of bleeding on probing (*p* = 0.02) and probing depth (*p* = 0.01). However, Bienz et al. in a retrospective study noticed that connective tissue grafts might be only marginally beneficial to prevent peri‐implant mucositis but not peri‐implantitis.^20^ In this sense, it is worth noting that in a prospective cohort study there appeared to be an adverse influence on peri‐implant health in patients with a history of periodontitis who had a thicker crestal soft tissue profile (OR = 2.52, 95% CI = 1.63–3.90, *p* < 0.0001).^38^ Surgical stage, whether its immediate, early or delay implant placement, did not yield a significant impact. However, Jemt et al. identified a risk 1.8x greater for immediate placement protocol when compared with other protocols used.^24^ Ortiz‐Echeverri et al. also demonstrated that immediate implants were more likely to be diagnosed with peri‐implantitis than those following delayed protocols (OR = 2.61, 95% CI = 1.4–5.1, *p* = 0.004).^36^ Moreover, bone augmentation aimed at restoring the atrophic alveolar ridge for accommodating a standard dental implant, was associated with the development of peri‐implantitis (OR = 2.35, 95% CI = 1.43–3.88, *p* = 0.0008).^21^ Moreover, implants placed into alveolar sites with limited width were also 2.9x more likely to develop peri‐implantitis (OR = 2.9, 95% CI = 1.8–4.6, *p* < 0.001).^36^ Further, non‐comparative studies suggested that guided implant surgery,^39^ immediate implants with immediate restoration^34^, ^35^ and narrower dental implants^31^ tended to achieve more favorable outcomes with low rates of biological complications.

#### 3.2.3 Risk of bias

Risk of bias are displayed in Table 1. Cross‐sectional, cohort and case series studies fulfilled ∼60% of the items. The item where most of them failed was sample size justification, blinding assessment, and uniformity to all participants. RCTs demonstrated low risk of bias.

### 3.3 Implant‐related factors and onset/progression of peri‐implant diseases

#### 3.3.1 Sample characteristics

The majority of included studies were RCTs (*n* = 5)^40^, ^41^, ^42^, ^43^, ^44^ followed by prospective cohort (*n* = 3)^45^, ^46^, ^47^ studies. Implant system was the most frequently analyzed implant‐related factor.^34^, ^42^, ^43^, ^47^, ^48^ The follow‐up times reported in the included studies ranged from 1 to 18 years. In total, 1192 patients and 4072 implants were assessed. While the most recent studies adopted the World Workshop AAP/EFP (2017) definition for peri‐implant diseases,^1^ studies prior to 2018 opted to adhere case definitions heterogeneous vastly based on bleeding on probing to define mucositis and bleeding on probing and/or suppuration and marginal bone loss in a range from 0.5 to 2  mm to define peri‐implantitis (Table 2).

#### 3.3.2 Impact on peri‐implant diseases

In general, data derived from comparative studies did not display superiority for any implant macro‐ or micro‐design characteristics.^34^, ^40^, ^41^, ^42^, ^43^, ^44^, ^46^, ^47^, ^49^ Marrone et al. in a cross‐sectional study, demonstrated that tissue‐level implants tended to be 1/3 less likely to have peri‐implantitis at the implant level compared with bone level implants (OR = 0.73, 95% CI = 0.25–2.22), although statistical significance was not reached.^50^ Another study observed that anodized and fluoride‐treated surface dental implants where 3.7x (OR = 3.77, 95% CI = 1.60‐8.87, *p* = 0.002) and 3.5x (OR = 3.55, 95% CI = 1.29–9.77, *p* = 0.014) more prone to exhibit peri‐implantitis compared with sand‐blast acid‐etched surface implants.^48^ This finding has not been confirmed by other studies and might be, in part, attributable to implant macro‐design since implants where the restorative margin was <1.5  mm from the alveolar bone at baseline demonstrated 2.3x (OR = 2.29, 95% CI = 1.21–4.34, *p* = 0.011) more peri‐implantitis when compared with restorative margins ≥1.5  mm from the alveolar bone (i.e., tissue level one piece implants or implants restored with a transmucosal abutment).^48^ Two prospective studies demonstrated that the use of a hybrid designed dental implant failed outperform the use of implants that were rough to the top.^40^, ^44^ Additionally, data regarding the use of laser‐microgrooved collar for the prevention of peri‐implant diseases was not conclusive. One study failed to prove that this feature was protective of peri‐implantitis,^49^ while a multi‐center retrospective trial showed ∼10% lower mucositis and peri‐implantitis at patient‐level.^51^

#### 3.3.3 Risk of bias

Risk of bias al displayed in Table 2. Cross‐sectional, and cohort studies fulfilled ∼70% of the items. The item where most of them failed was blinding assessment and uniformity to all participants. Overall, 25% of the RCTs demonstrated low risk of bias, while 75% showed concerns related to blinding during assessment.

## 4 DISCUSSION

The site‐specificity pattern of peri‐implantitis suggests that there are local factors that not only contribute to its onset but also influence the progression of this disease. It is worth noting, that peri‐implant mucositis appears to always precede peri‐implantitis, even though the prevalence seems to be equivalent at patient‐ and implant‐level. Hence, the primary and secondary prevention are keys to achieve long‐term hard and soft tissue stability. In this sense, the identification and modification, whenever present, of local predisposing/precipitating factors would be paramount to prevent biofilm formation and maturation. Considering the concerns raised in the effectiveness of non‐surgical and surgical measures to arrest peri‐implantitis, the clinician is advised to make every effort possible to minimize the risk of peri‐implant diseases. The objective of this systematic review was to provide insight into the role of surgical‐ and implant‐related factors on the onset/progression of peri‐implantitis. Modest clinical evidence appears to be present for surgical‐factors, in particular inadequate implant position and, to a lesser extent, implants placed in regenerated bone, are more prone to exhibit peri‐implantitis (Figure 2c), but not peri‐implant mucositis. One must bear in mind that this may be due to studies failing to look at this association leading to a lack of evidence. However, this systematic review failed to identify any clinical evidence on the superiority of a specific implant macro‐ or micro‐design that achieves. Nonetheless, some of the evidence would suggest that a shortened distance from the prosthetic margin to crestal bone during implant placement may predispose peri‐implantitis. This would appear to favor the concept of a tissue level‐oriented implant design or the use of transmucosal abutments.

Emerging data suggested the crucial role of local predisposing factors in the onset/progression of peri‐implant diseases.^15^ Conceptually, predisposing factors refer to conditions that places the given element (dental implant)/individual(patient) at risk of developing a problem (peri‐implant disease).^14^ In particular, during the surgical phase, many factors have to be orchestrated in anticipating bone apposition along the implant surface to minimize biofilm attachment in the post‐operative (short‐ and long‐term) phase. Otherwise, the exposed implant surface represents a suitable niche for biofilm formation and maturation. Implants are in certain circumstances placed with a suboptimal three‐dimensional position. One example is displayed by when the drilling protocol is insufficient and the roughened surface of the coronal portion is left exposed to the supra‐crestal environment. Another archetype is when implants are placed outside of the bony housing due to the reduced ridge dimension,^36^ increased implant width^31^ or “too‐buccal” implant placement (Figure 2d,e).^22^ While bone regeneration efforts may have been successful to increase ridge width, the inevitable success to integrate resides with alveolar ridge morphology, in particular to the implant position in relation with the alveolar contour.^52^, ^53^ This may explain why failed bone regeneration sites tend to experience 2.3x higher risk of peri‐implantitis.^21^ While bone regeneration may recapture some of the bone lost and the implant may be appropriately positioned within this site, bone remodeling on the facial may work against maintaining ridge with in these sites. One cannot overlook that bone response/remodeling is dominantly governed by bone characteristics. In this sense, it is critical to understand that bone is formed by two different compartments. The cortical layer, located at the outer side of the alveolar bone, receives blood supply from the periosteum. When an implant is inserted with an open‐flap approach, elevation of the periosteum eliminates the periosteal blood supply from the outside. If the underlying bone fails to have an adequate endosteal marrow space, to support its maintenance, there will be crestal die back. The same process occurs from the inside, since insertion of the implant interrupts the endosteal blood supply, bone maintenance relies solely on the periosteal blood supply. This may lead to a physiological phenomenon process known as “avascular necrosis”^54^ where the bone reabsorbs in a horizontal fashion and leaves the implant surface expose to the peri‐implant sulcus where surface contamination is likely to occur. Monje et al. demonstrated in a preclinical study the role of the buccal bone thickness to prevent major physiological and pathological bone loss.^55^ These authors saw that when the buccal bone was <1.5  mm in thickness, there was, on average, approximately 4  mm of buccal bone loss which might facilitate surface contamination and subsequent inflammation/disease establishment.^55^ This observation has been supported by clinical studies included in a systematic review.^56^ This finding may further explain why the majority of peri‐implantitis diagnosed exhibit a 2/3‐wall defect configuration where the buccal wall is often missing.^57^ The present systematic review identified several papers that highlighted the role of implant malposition on the onset/progression of peri‐implantitis.^21^, ^22^, ^26^, ^29^ To overcome these situations, data suggested that implant placements must take into account proper positioning to provide proper prosthetics where emergence profile^58^ is critical and surgical principles to maintain adequate bone dimensions to surgically induced implant surface exposure. Possible consideration needs to be given to performing proactive phenotype modification with guided bone regeneration and possibly soft tissue grafting to maximize tissue stability following implant placement.^59^, ^60^

Additionally, inadequate apico‐coronal implant position has been suggested to contribute to peri‐implant mucositis^32^ and peri‐implantitis.^26^ Kumar et al. in a retrospective analysis of non‐splinted restorations found that implants placed at an apical level ≥6  mm from the adjacent cemento‐enamel junctions had a 8.5× higher risk of being diagnosed with peri‐implantitis.^26^ Broggini et al. demonstrated that the implant‐abutment interface dictates the intensity and location of peri‐implant inflammatory cell accumulation as a subcrestal interface promoted a significantly greater invasion of neutrophils than did supra‐crestal interfaces (10512 vs. 2398 neutrophils/mm^2^).^61^ This inflammatory infiltrate manifests itself with pathologic hard and soft tissue instability (Figure 2f). This agrees with Chan et al. who reported that deeper positioned implants (≥3  mm) were more closely linked to the ineffective resolution of experimental mucositis.^62^ To overcome these situations, use of transmucosal abutments (≥2  mm) may be effective to prevent marginal bone.^63^, ^64^ Interestingly, this systematic review identified one prospective cohort that failed to show that thicker soft tissues at the crestal aspect are beneficial to prevent peri‐implant diseases.^38^ Therefore, soft tissue grafting to thicken the connective tissue might be a strategy to enhance esthetics at the buccal aspect but has not been proven to effectively prevent peri‐implant disease whenever used in the crestal area.

**FIGURE 3:**
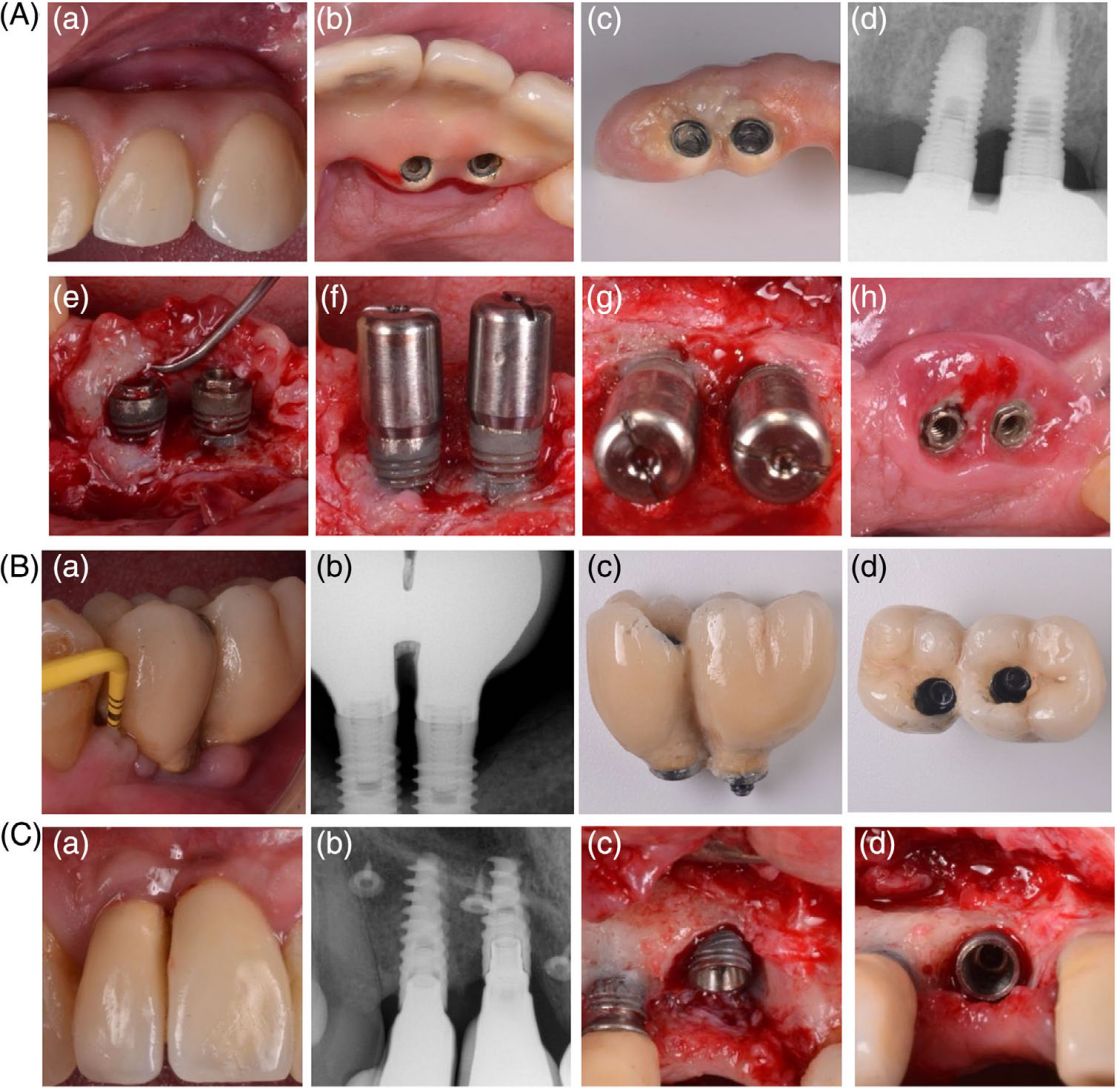
(A) Peri‐implantitis‐related supra‐crestal defects are often associated to implants very close to each other. (a) Frontal view, (b) occlusal view, (c) prosthesis design precludes self‐performed oral hygiene care, (d) periapical radiographic shows supra‐crestal defect, (e) clinical signs of inflammation, (f) curettage is performed to gain access, (g) frontal view of implants very close to each other, (h) occlusal view of malpositioned implants. (B) Implant proximity was suggested being predisposing of peri‐implantitis. (a) Clinical image showing signs of inflammation, (b) periapical x‐ray illustrating implant proximity and peri‐implantitis‐related bone loss, (c) prosthesis design due to implant proximity precludes effective proximal self‐performed oral hygiene measures, (d) occlusal view of the prosthesis. (C) Vertical discrepancies of implants are often associated with peri‐implantitis. (a) Clinical signs of inflammation, (b) periapical x‐ray illustrating mild peri‐implantitis‐related bone loss, (c) frontal view of peri‐implantitis‐related bone defect, (d) occlusal view of peri‐implantitis‐related bone defect.

Another factor found to be related to peri‐implantitis is improper implant position from a mesio‐buccal perspective.^29^ Schuldt‐Filho et al. demonstrated that implants placed in close proximity to one another (<3  mm) were 8.6× more exposed to have peri‐implantitis when compared with implants placed ≥3  mm apart (Figure 3a,b).^29^ This finding might be attributable to limited interproximal space does not favor proper proximal self‐performed oral hygiene measures^65^ or to physiologic bone loss related to violating blood supply.^66^ Monje et al. further noticed that teeth adjacent to untreated peri‐implantitis lesions are associated with proximal loss of periodontal support, being more remarkable in scenarios that display short implant‐tooth distance.^67^ Lin et al. showed that two or three adjacent implants placed with a vertical platform discrepancy are associated with higher incidence of ≥1 mm marginal bone loss (Figure 3c).^68^ This scenario may favor an anaerobic ecosystem that can contribute to the onset of peri‐implantitis. Contrary to this, Corbella et al. failed to identify implant position as contributor to peri‐implantitis. This might be due to the nature of the study and the vague definition of implant malposition based on periapical radiographs.^23^ Again, implant position must consider prosthetic guidance and not just by mesio‐distal dimension. In this sense, narrow diameter implants demonstrated more favorable outcomes.^69^ Nevertheless, their use is subjected to the most suitable emergence profile according to the anatomical region.

**FIGURE 4:**
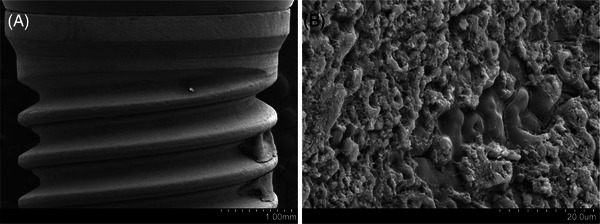
Bacterial biofilm is the etiologic factor of peri‐implant diseases. (A,B) Macro‐ and micro‐structural topographic irregularities favor the adhesion of biofilm.

Implant‐related factors were explored and comparative studies failed to reveal any implant feature that increases the risk to developing peri‐implant diseases (Figure 4). Interestingly, Marrone et al. in a cross‐sectional study demonstrated that tissue‐level implants tended to display about 1/3× less peri‐implantitis at the implant‐level when compared with bone‐level implants.^50^ This is in line with another study whom showed that anodized and fluoride‐treated surface dental implants where 3.7× and 3.5× more associated to peri‐implantitis compared with sand‐blast acid‐etched surface implants.^48^ The tissue level feature of the implants in this study may have accounted for why those other implants where the distance from the prosthetic margin to crestal bone at baseline was ≤1.5  mm were at greater risk, 2.3×, for peri‐implantitis.^48^ Hence it would appear that using a tissue level design or transmucosal abutments might be more protective from developing peri‐implantitis.

It is relevant, though, to point out that preclinical studies have further focused toward the role of implant surface topographic characteristics on peri‐implantitis. Ligature‐induced peri‐implantitis studies have suggested that a roughened anodized implant surface exhibits faster progressing peri‐implant bone loss when compared with other implant surfaces/systems and in particular to one that is machined.^70^, ^71^, ^72^, ^73^ Hence, this points towards the incorporation of hybrid surfaces to minimize surface roughness at the coronal portion of dental implants. Serrano et al. demonstrated that marginal bone level was equal to completely rough implants.^40^ The hypothesis behind their use is that, if exposed due to major physiological or pathological bone loss, bacterial adhesion might be challenged while fibroblast adhesion and homeostasis may enhance the sealing of the peri‐implant compartment.^74^ Monje et al. showed in a ligature‐induced peri‐implantitis model that, during spontaneous progression, hybrid design implants displayed statistically significant lower levels of IL‐1β compared with completely rough implants.^8^ This indicates a lesser inflammatory activity of hybrid implants which, in turn, may assist in arresting progressive bone loss in the clinical basis. Nevertheless, Gilbert et al. failed to show the outperformance of hybrid design implants when compared with moderately rough implants in a 5‐year split mouth study of patients under supportive care.^44^ Hence, their potential might be disclosed in susceptible patients due to the presence of systemic drivers, unhealthy habits or erratic compliance with supportive care. For instance, Sicilia et al. noticed less crestal bone loss of hybrid and turned implants when compared with anodized implants in patients in history of periodontitis. Along these lines, it is relevant to point out that the exposure of the surface microroughness to the oral cavity or the peri‐implant sulcus seems conducive to peri‐implantitis.^75^ Windael et al. demonstrated that implants with early bone loss ≥0.5 mm during the first year of function showed a 5× higher odds for future peri‐implantitis development (study period = 10 years).^76^ This indicates that measures must be adopted in selecting the most suitable implant design and prosthetic components according to the case scenario. This is in agreement with data published elsewhere.^77^, ^78^

## 5 CONCLUSIONS

Considering the existing evidence and their underlying study design and the quality of the retrieved data, we noted that modest data suggest that certain surgical‐related and implant‐related factors are associated with the onset/progression of peri‐implant diseases. In particular, implant malposition seems to have a notable impact on peri‐implantitis. In addition, it seems that a short distance from the prosthetic margin to crestal bone during implant placement may predispose peri‐implantitis. This would favor the concept of tissue‐level implant design or the use of transmucosal abutments.

## AUTHOR CONTRIBUTIONS

All the authors conceived the concept. Alberto Monje and Shayan Barootchi performed the screening. Paul S. Rosen and Hom‐Lay Wang supervised the writing.

## CONFLICT OF INTEREST STATEMENT

None of the authors disclose a direct conflict of interest.

## FUNDING INFORMATION

The authors received no specific funding for this work.

## Supporting information



Supporting information

## Data Availability

The data that support the findings of this study are available from the corresponding author upon reasonable request.
