# Supplementary material for: Surgical‐ and implant‐related factors and onset/progression of peri‐implant diseases: An AO/AAP systematic review
Source: J Periodontol. 2025 Jun 9;96(6):542–61. doi: 10.1002/JPER.24-0083 (PMC12273751; doi:10.1002/JPER.24-0083)
Supplement: Supplementary file 1 — Supporting information [file JPER-96-542-s001.docx]

| **Supplementary table 1.** Screening approach for the surgical- and implant-related factors associated with peri-implant diseases | | | |
| --- | --- | --- | --- |
| **Factor** |  | **Mucositis** | **Peri-implantitis** |
| **Surgical-related** | **Implant position** | ("drug implants"[MeSH Terms] OR ("drug"[All Fields] AND "implants"[All Fields]) OR "drug implants"[All Fields] OR "implant"[All Fields] OR "embryo implantation"[MeSH Terms] OR ("embryo"[All Fields] AND "implantation"[All Fields]) OR "embryo implantation"[All Fields] OR "implantation"[All Fields] OR "implant s"[All Fields] OR "implantability"[All Fields] OR "implantable"[All Fields] OR "implantables"[All Fields] OR "implantate"[All Fields] OR "implantated"[All Fields] OR "implantates"[All Fields] OR "implantations"[All Fields] OR "implanted"[All Fields] OR "implanter"[All Fields] OR "implanters"[All Fields] OR "implanting"[All Fields] OR "implantion"[All Fields] OR "implantitis"[All Fields] OR "implants"[All Fields]) AND ("patient positioning"[MeSH Terms] OR ("patient"[All Fields] AND "positioning"[All Fields]) OR "patient positioning"[All Fields] OR "positioning"[All Fields] OR "position"[All Fields] OR "position s"[All Fields] OR "positional"[All Fields] OR "positioned"[All Fields] OR "positionings"[All Fields] OR "positions"[All Fields]) AND "peri-implant"[All Fields] AND ("mucosalization"[All Fields] OR "mucosalized"[All Fields] OR "mucosally"[All Fields] OR "mucose"[All Fields] OR "mucoses"[All Fields] OR "mucositis"[MeSH Terms] OR "mucositis"[All Fields] OR "mucositides"[All Fields] OR "mucous membrane"[MeSH Terms] OR ("mucous"[All Fields] AND "membrane"[All Fields]) OR "mucous membrane"[All Fields] OR "mucosal"[All Fields]) | ("drug implants"[MeSH Terms] OR ("drug"[All Fields] AND "implants"[All Fields]) OR "drug implants"[All Fields] OR "implant"[All Fields] OR "embryo implantation"[MeSH Terms] OR ("embryo"[All Fields] AND "implantation"[All Fields]) OR "embryo implantation"[All Fields] OR "implantation"[All Fields] OR "implant s"[All Fields] OR "implantability"[All Fields] OR "implantable"[All Fields] OR "implantables"[All Fields] OR "implantate"[All Fields] OR "implantated"[All Fields] OR "implantates"[All Fields] OR "implantations"[All Fields] OR "implanted"[All Fields] OR "implanter"[All Fields] OR "implanters"[All Fields] OR "implanting"[All Fields] OR "implantion"[All Fields] OR "implantitis"[All Fields] OR "implants"[All Fields]) AND ("patient positioning"[MeSH Terms] OR ("patient"[All Fields] AND "positioning"[All Fields]) OR "patient positioning"[All Fields] OR "positioning"[All Fields] OR "position"[All Fields] OR "position s"[All Fields] OR "positional"[All Fields] OR "positioned"[All Fields] OR "positionings"[All Fields] OR "positions"[All Fields]) AND ("peri implantitis"[MeSH Terms] OR "peri implantitis"[All Fields] OR ("peri"[All Fields] AND "implantitis"[All Fields]) OR "peri implantitis"[All Fields]) |
|  | **Result** | 123 | 176 |
|  | **Surgical phase** | ("drug implants"[MeSH Terms] OR ("drug"[All Fields] AND "implants"[All Fields]) OR "drug implants"[All Fields] OR "implant"[All Fields] OR "embryo implantation"[MeSH Terms] OR ("embryo"[All Fields] AND "implantation"[All Fields]) OR "embryo implantation"[All Fields] OR "implantation"[All Fields] OR "implant s"[All Fields] OR "implantability"[All Fields] OR "implantable"[All Fields] OR "implantables"[All Fields] OR "implantate"[All Fields] OR "implantated"[All Fields] OR "implantates"[All Fields] OR "implantations"[All Fields] OR "implanted"[All Fields] OR "implanter"[All Fields] OR "implanters"[All Fields] OR "implanting"[All Fields] OR "implantion"[All Fields] OR "implantitis"[All Fields] OR "implants"[All Fields]) AND ("placement"[All Fields] OR "placements"[All Fields]) AND ("mucosalization"[All Fields] OR "mucosalized"[All Fields] OR "mucosally"[All Fields] OR "mucose"[All Fields] OR "mucoses"[All Fields] OR "mucositis"[MeSH Terms] OR "mucositis"[All Fields] OR "mucositides"[All Fields] OR "mucous membrane"[MeSH Terms] OR ("mucous"[All Fields] AND "membrane"[All Fields]) OR "mucous membrane"[All Fields] OR "mucosal"[All Fields]) | ("drug implants"[MeSH Terms] OR ("drug"[All Fields] AND "implants"[All Fields]) OR "drug implants"[All Fields] OR "implant"[All Fields] OR "embryo implantation"[MeSH Terms] OR ("embryo"[All Fields] AND "implantation"[All Fields]) OR "embryo implantation"[All Fields] OR "implantation"[All Fields] OR "implant s"[All Fields] OR "implantability"[All Fields] OR "implantable"[All Fields] OR "implantables"[All Fields] OR "implantate"[All Fields] OR "implantated"[All Fields] OR "implantates"[All Fields] OR "implantations"[All Fields] OR "implanted"[All Fields] OR "implanter"[All Fields] OR "implanters"[All Fields] OR "implanting"[All Fields] OR "implantion"[All Fields] OR "implantitis"[All Fields] OR "implants"[All Fields]) AND ("placement"[All Fields] OR "placements"[All Fields]) AND ("peri implantitis"[MeSH Terms] OR "peri implantitis"[All Fields] OR ("peri"[All Fields] AND "implantitis"[All Fields]) OR "peri implantitis"[All Fields]) |
|  | **Result** | 847 | 505 |
|  | **Implant placement protocol** | ("drug implants"[MeSH Terms] OR ("drug"[All Fields] AND "implants"[All Fields]) OR "drug implants"[All Fields] OR "implant"[All Fields] OR "embryo implantation"[MeSH Terms] OR ("embryo"[All Fields] AND "implantation"[All Fields]) OR "embryo implantation"[All Fields] OR "implantation"[All Fields] OR "implant s"[All Fields] OR "implantability"[All Fields] OR "implantable"[All Fields] OR "implantables"[All Fields] OR "implantate"[All Fields] OR "implantated"[All Fields] OR "implantates"[All Fields] OR "implantations"[All Fields] OR "implanted"[All Fields] OR "implanter"[All Fields] OR "implanters"[All Fields] OR "implanting"[All Fields] OR "implantion"[All Fields] OR "implantitis"[All Fields] OR "implants"[All Fields]) AND ("placement"[All Fields] OR "placements"[All Fields]) AND ("protocol"[All Fields] OR "protocol s"[All Fields] OR "protocolized"[All Fields] OR "protocols"[All Fields]) AND ("mucosalization"[All Fields] OR "mucosalized"[All Fields] OR "mucosally"[All Fields] OR "mucose"[All Fields] OR "mucoses"[All Fields] OR "mucositis"[MeSH Terms] OR "mucositis"[All Fields] OR "mucositides"[All Fields] OR "mucous membrane"[MeSH Terms] OR ("mucous"[All Fields] AND "membrane"[All Fields]) OR "mucous membrane"[All Fields] OR "mucosal"[All Fields]) | ("drug implants"[MeSH Terms] OR ("drug"[All Fields] AND "implants"[All Fields]) OR "drug implants"[All Fields] OR "implant"[All Fields] OR "embryo implantation"[MeSH Terms] OR ("embryo"[All Fields] AND "implantation"[All Fields]) OR "embryo implantation"[All Fields] OR "implantation"[All Fields] OR "implant s"[All Fields] OR "implantability"[All Fields] OR "implantable"[All Fields] OR "implantables"[All Fields] OR "implantate"[All Fields] OR "implantated"[All Fields] OR "implantates"[All Fields] OR "implantations"[All Fields] OR "implanted"[All Fields] OR "implanter"[All Fields] OR "implanters"[All Fields] OR "implanting"[All Fields] OR "implantion"[All Fields] OR "implantitis"[All Fields] OR "implants"[All Fields]) AND ("placement"[All Fields] OR "placements"[All Fields]) AND ("protocol"[All Fields] OR "protocol s"[All Fields] OR "protocolized"[All Fields] OR "protocols"[All Fields]) AND ("peri implantitis"[MeSH Terms] OR "peri implantitis"[All Fields] OR ("peri"[All Fields] AND "implantitis"[All Fields]) OR "peri implantitis"[All Fields]) |
|  | **Result** | 101 | 73 |
|  | **Soft tissue grafting** | "soft"[All Fields] AND ("tissue transplantation"[MeSH Terms] OR ("tissue"[All Fields] AND "transplantation"[All Fields]) OR "tissue transplantation"[All Fields] OR ("tissue"[All Fields] AND "grafting"[All Fields]) OR "tissue grafting"[All Fields]) AND ("mucosalization"[All Fields] OR "mucosalized"[All Fields] OR "mucosally"[All Fields] OR "mucose"[All Fields] OR "mucoses"[All Fields] OR "mucositis"[MeSH Terms] OR "mucositis"[All Fields] OR "mucositides"[All Fields] OR "mucous membrane"[MeSH Terms] OR ("mucous"[All Fields] AND "membrane"[All Fields]) OR "mucous membrane"[All Fields] OR "mucosal"[All Fields]) | "soft"[All Fields] AND ("tissue transplantation"[MeSH Terms] OR ("tissue"[All Fields] AND "transplantation"[All Fields]) OR "tissue transplantation"[All Fields] OR ("tissue"[All Fields] AND "grafting"[All Fields]) OR "tissue grafting"[All Fields]) AND ("peri implantitis"[MeSH Terms] OR "peri implantitis"[All Fields] OR ("peri"[All Fields] AND "implantitis"[All Fields]) OR "peri implantitis"[All Fields]) |
|  | **Result** | 458 | 56 |
| **Implant-related** | **Implant macro-geometry (system)** | ("drug implants"[MeSH Terms] OR ("drug"[All Fields] AND "implants"[All Fields]) OR "drug implants"[All Fields] OR "implant"[All Fields] OR "embryo implantation"[MeSH Terms] OR ("embryo"[All Fields] AND "implantation"[All Fields]) OR "embryo implantation"[All Fields] OR "implantation"[All Fields] OR "implant s"[All Fields] OR "implantability"[All Fields] OR "implantable"[All Fields] OR "implantables"[All Fields] OR "implantate"[All Fields] OR "implantated"[All Fields] OR "implantates"[All Fields] OR "implantations"[All Fields] OR "implanted"[All Fields] OR "implanter"[All Fields] OR "implanters"[All Fields] OR "implanting"[All Fields] OR "implantion"[All Fields] OR "implantitis"[All Fields] OR "implants"[All Fields]) AND ("system"[All Fields] OR "system s"[All Fields] OR "systems"[All Fields]) AND "peri-implant"[All Fields] AND ("mucosalization"[All Fields] OR "mucosalized"[All Fields] OR "mucosally"[All Fields] OR "mucose"[All Fields] OR "mucoses"[All Fields] OR "mucositis"[MeSH Terms] OR "mucositis"[All Fields] OR "mucositides"[All Fields] OR "mucous membrane"[MeSH Terms] OR ("mucous"[All Fields] AND "membrane"[All Fields]) OR "mucous membrane"[All Fields] OR "mucosal"[All Fields]) | ("drug implants"[MeSH Terms] OR ("drug"[All Fields] AND "implants"[All Fields]) OR "drug implants"[All Fields] OR "implant"[All Fields] OR "embryo implantation"[MeSH Terms] OR ("embryo"[All Fields] AND "implantation"[All Fields]) OR "embryo implantation"[All Fields] OR "implantation"[All Fields] OR "implant s"[All Fields] OR "implantability"[All Fields] OR "implantable"[All Fields] OR "implantables"[All Fields] OR "implantate"[All Fields] OR "implantated"[All Fields] OR "implantates"[All Fields] OR "implantations"[All Fields] OR "implanted"[All Fields] OR "implanter"[All Fields] OR "implanters"[All Fields] OR "implanting"[All Fields] OR "implantion"[All Fields] OR "implantitis"[All Fields] OR "implants"[All Fields]) AND ("system"[All Fields] OR "system s"[All Fields] OR "systems"[All Fields]) AND ("peri implantitis"[MeSH Terms] OR "peri implantitis"[All Fields] OR ("peri"[All Fields] AND "implantitis"[All Fields]) OR "peri implantitis"[All Fields]) |
|  | **Result** | 115 | 393 |
|  | **Implant surface** | ("drug implants"[MeSH Terms] OR ("drug"[All Fields] AND "implants"[All Fields]) OR "drug implants"[All Fields] OR "implant"[All Fields] OR "embryo implantation"[MeSH Terms] OR ("embryo"[All Fields] AND "implantation"[All Fields]) OR "embryo implantation"[All Fields] OR "implantation"[All Fields] OR "implant s"[All Fields] OR "implantability"[All Fields] OR "implantable"[All Fields] OR "implantables"[All Fields] OR "implantate"[All Fields] OR "implantated"[All Fields] OR "implantates"[All Fields] OR "implantations"[All Fields] OR "implanted"[All Fields] OR "implanter"[All Fields] OR "implanters"[All Fields] OR "implanting"[All Fields] OR "implantion"[All Fields] OR "implantitis"[All Fields] OR "implants"[All Fields]) AND ("surface"[All Fields] OR "surface s"[All Fields] OR "surfaced"[All Fields] OR "surfaces"[All Fields] OR "surfacing"[All Fields] OR "surfacings"[All Fields]) AND "peri-implant"[All Fields] AND ("mucosalization"[All Fields] OR "mucosalized"[All Fields] OR "mucosally"[All Fields] OR "mucose"[All Fields] OR "mucoses"[All Fields] OR "mucositis"[MeSH Terms] OR "mucositis"[All Fields] OR "mucositides"[All Fields] OR "mucous membrane"[MeSH Terms] OR ("mucous"[All Fields] AND "membrane"[All Fields]) OR "mucous membrane"[All Fields] OR "mucosal"[All Fields]) | ("drug implants"[MeSH Terms] OR ("drug"[All Fields] AND "implants"[All Fields]) OR "drug implants"[All Fields] OR "implant"[All Fields] OR "embryo implantation"[MeSH Terms] OR ("embryo"[All Fields] AND "implantation"[All Fields]) OR "embryo implantation"[All Fields] OR "implantation"[All Fields] OR "implant s"[All Fields] OR "implantability"[All Fields] OR "implantable"[All Fields] OR "implantables"[All Fields] OR "implantate"[All Fields] OR "implantated"[All Fields] OR "implantates"[All Fields] OR "implantations"[All Fields] OR "implanted"[All Fields] OR "implanter"[All Fields] OR "implanters"[All Fields] OR "implanting"[All Fields] OR "implantion"[All Fields] OR "implantitis"[All Fields] OR "implants"[All Fields]) AND ("surface"[All Fields] OR "surface s"[All Fields] OR "surfaced"[All Fields] OR "surfaces"[All Fields] OR "surfacing"[All Fields] OR "surfacings"[All Fields]) AND ("peri implantitis"[MeSH Terms] OR "peri implantitis"[All Fields] OR ("peri"[All Fields] AND "implantitis"[All Fields]) OR "peri implantitis"[All Fields]) |
|  | **Result** | 250 | 1189 |
